# Ex vivo SARS-CoV-2 infection of human lung reveals heterogeneous host defense and therapeutic responses

**DOI:** 10.1172/jci.insight.148003

**Published:** 2021-09-22

**Authors:** Matthew A. Schaller, Yamini Sharma, Zadia Dupee, Duy Nguyen, Juan Urueña, Ryan Smolchek, Julia C. Loeb, Tiago N. Machuca, John A. Lednicky, David J. Odde, Robert F. Campbell, W. Gregory Sawyer, Borna Mehrad

**Affiliations:** 1 Division of Pulmonary, Critical Care, and Sleep Medicine, Department of Medicine, College of Medicine;; 2 Department of Mechanical and Aerospace Engineering, Herbert Wertheim College of Engineering;; 3 Department of Environmental and Global Health, College of Public Health and Health Professions, and Emerging Pathogens Institute; and; 4 Division of Cardiothoracic Surgery, College of Medicine, University of Florida, Gainesville, Florida, USA.; 5 Department of Biomedical Engineering, College of Science and Engineering, University of Minnesota, Minneapolis, Minnesota, USA.; 6 Department of Drug Development, Walter Reed Army Institute of Research, Silver Spring, Maryland, USA.

**Keywords:** COVID-19, Inflammation, Cellular immune response, Drug screens

## Abstract

Cell lines are the mainstay in understanding the biology of COVID-19 infection but do not recapitulate many of the complexities of human infection. The use of human lung tissue is one solution for the study of such novel respiratory pathogens. We hypothesized that a cryopreserved bank of human lung tissue would allow for the ex vivo study of the interindividual heterogeneity of host response to SARS-CoV-2, thus providing a bridge between studies with cell lines and studies in animal models. We generated a cryobank of tissues from 21 donors, many of whom had clinical risk factors for severe COVID-19. Cryopreserved tissues preserved 90% cell viability and contained heterogenous populations of metabolically active epithelial, endothelial, and immune cell subsets of the human lung. Samples were readily infected with HCoV-OC43 and SARS-CoV-2 and demonstrated comparable susceptibility to infection. In contrast, we observed a marked donor-dependent heterogeneity in the expression of *IL6*, *CXCL8*, and *IFNB1* in response to SARS-CoV-2. Treatment of tissues with dexamethasone and the experimental drug N-hydroxycytidine suppressed viral growth in all samples, whereas chloroquine and remdesivir had no detectable effect. Metformin and sirolimus, molecules with predicted but unproven antiviral activity, each suppressed viral replication in tissues from a subset of donors. In summary, we developed a system for the ex vivo study of human SARS-CoV-2 infection using primary human lung tissue from a library of donor tissues. This model may be useful for drug screening and for understanding basic mechanisms of COVID-19 pathogenesis.

## Graphical abstract

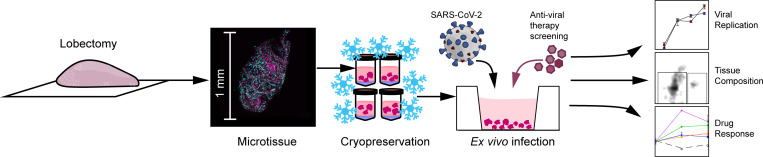

## Introduction

The COVID-19 pandemic has highlighted the need for improved models of infection to study host-pathogen interactions, rapidly screen potential therapeutic interventions, and study fundamental pathogenic mechanisms. Cell lines have served as the mainstay in understanding the biology of the infection but do not capture the cellular heterogeneity, cell-cell and cell-matrix interactions, or between-host variability encountered in the human infection.

The use of human lung tissue is one solution for the study of biology of novel respiratory pathogens: lung tissue explants capture the cellular heterogeneity within the human lung, and the use of tissues from multiple donors can allow the study of the genetic diversity found within the human population. To this end, human lung organoids (1, 2) and lung-on-a-chip systems (3) can be readily infected with SARS-CoV-2. The use of these models is limited by 2 considerations: first, there are practical constraints on repeating experiments with tissue from a given donor at a later time or performing experiments with samples from multiple donors simultaneously. Second, these are simplified systems that do not fully recapitulate the complexities of lung tissue. The optimal solution to these problems is a repeatable and scalable in vitro system that captures the intricacies of the human lung and allows parallel testing with tissue from multiple donors in a single experiment and sequential experiments using tissue from the same donor.

Polyampholytes are polymeric hydrogels composed of poly-l-lysine or other amphiphilic chemicals that can serve as a less toxic alternative to DMSO for the cryopreservation of cells. Similar to antifreeze proteins first discovered in arctic fish (4), these amphiphilic proteins interact with both the hydrophobic cell membrane and hydrophilic ice crystals, mitigating damage associated with the formation of the crystals during freezing (5–7). Polyampholyte media have been used to cryopreserve many cell lines and primary cells with high viability (8). Recent work with polyampholytes has demonstrated that they can also be used to cryopreserve 3D cell structures (9). We therefore tested the hypothesis that a cryopreserved bank of human lung tissue allows for the ex vivo study of the interindividual heterogeneity of host response to SARS-CoV-2 infection, thus providing a bridge between studies with cell lines and studies in animal models.

## Results

We recruited 21 participants, most of whom had at least 1 risk factor for severe COVID-19 infection, including old age and comorbidities (Table 1).

### Composition of cryopreserved lung tissue.

**Figure 1:**
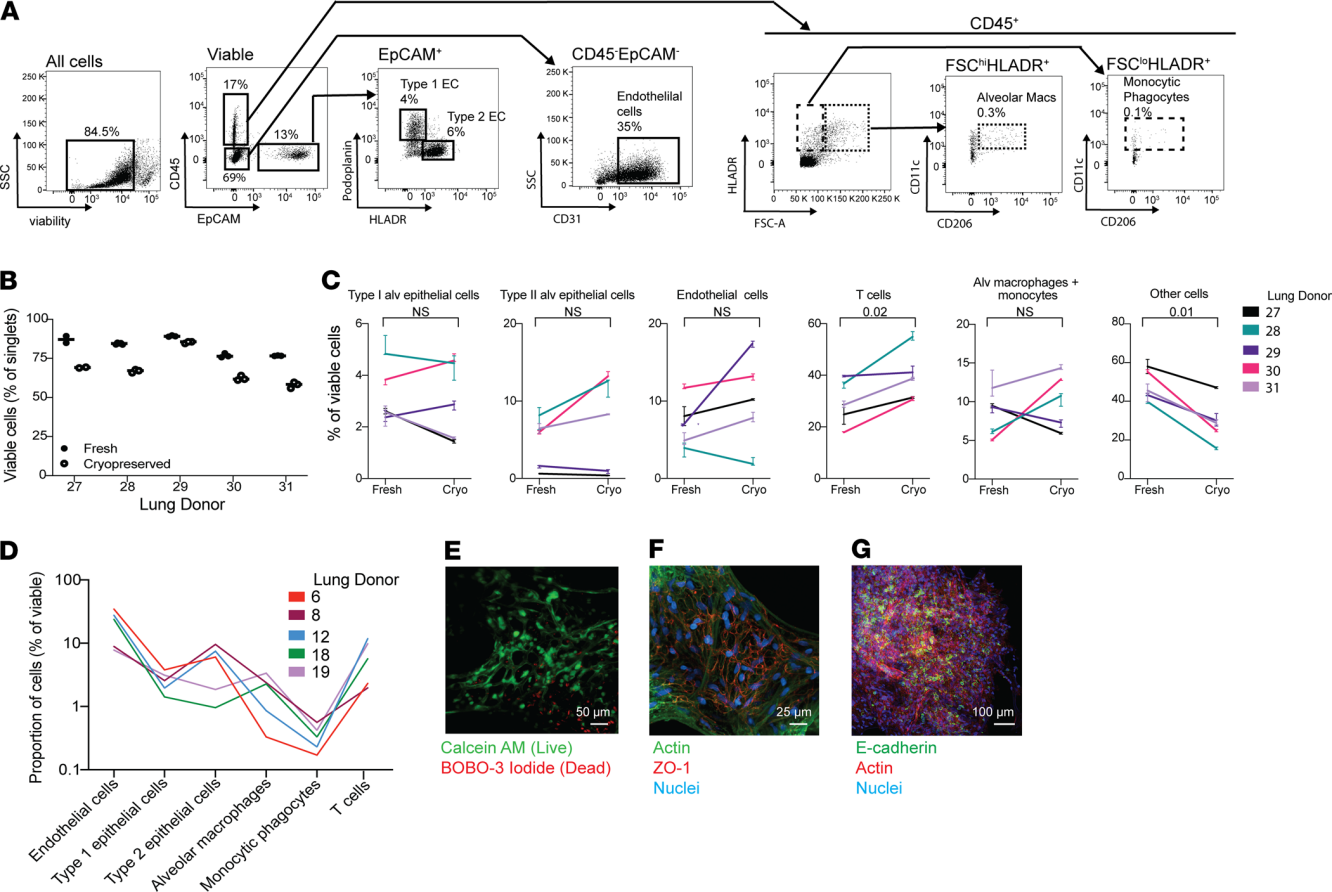
Lung microtissues are viable after cryopreservation. (**A**) The composition of cryopreserved lung was assessed by flow cytometry. Measured populations include type I and type II alveolar epithelial cells, endothelial cells, monocytes/macrophages, and T cell populations. (**B**) Proportion of viable cells in fresh and cryopreserved lung tissue from 5 donors. Samples were run in duplicate before and after cryopreservation. (**C**) The cellular composition of lung tissue, with a focus on the cell types depicted in **A** before and after cryopreservation for each of the 5 donors in **B**. Each line represents the average population present in 2–3 samples, consisting of 20–40 microtissues, from each donor. Error bars indicate variation in the technical replicates for each donor. (**D**) The cellular composition of cryopreserved samples from an additional 5 donors (separate from those in **A** and **B**) was assessed using the gating strategy depicted in **C**. (**E**) Viability of cryopreserved tissues was assessed by microscopy using Calcein AM and BOBO-3 iodide in microtissues cultured for 48 hours. Scale bar: 50 μm. (**F**) Tight junctions in cultured microtissues were also assessed using ZO-1 with costaining for actin and DAPI. Scale bar: 25 μm. (**G**) Microtissues stained with E-cadherin demonstrate the presence of epithelial cell populations within the cryopreserved samples. Scale bar: 100 μm.****

We began by assessing the viability and integrity of cryopreserved lung tissue, comparing viable populations of cells in cryopreserved tissues from the same donor and using a heat-killed control to determine the viability gate (Figure 1A and Supplemental Figure 1; supplemental material available online with this article; https://doi.org/10.1172/jci.insight.148003DS1). There was an approximately 15% decrease in overall viability of cryopreserved cells regardless of donor (Figure 1B), but viable populations of all major subsets of cells were present in cryopreserved tissue, including epithelial cells, endothelial cells, and leukocytes (Figure 1, C and D). Monocytic phagocytes composed approximately 0.1% of the total cell population, and alveolar macrophages made up approximately 0.5%. Each population, determined as a percentage of total, was consistent with previous results in which a collagenase-based method was used to digest the lung (10). Although there were no significant changes in most cell populations as a result of cryopreservation, we did observe a marked decrease in cells that were not identified by our flow panel, which may have included ciliated epithelial cells and fibroblasts. This was accompanied by a substantial increase in viable T cells, which likely survive the cryopreservation process at higher rates than other cell types and are overrepresented in the thawed sample. Flow cytometric analysis showed type I alveolar epithelial cells to constitute approximately 4% of total viable cells in both fresh and frozen tissue, lower than approximately 8% of lung tissue reported in the literature (11, 12). We attribute this to the loss of these fragile cells during processing for flow cytometry (13). To obtain a more accurate measurement of epithelial cell viability, we imaged the samples using confocal microscopy after staining with Calcein AM, a cell-permeable viability dye that only fluoresces in metabolically active cells, and found an abundant population of viable cells with type I epithelial morphology within the samples (Figure 1E). We confirmed this finding by staining the cryopreserved samples with zona occludens-1 (ZO-1), which identifies intact tight junctions between lung epithelial cells (14), and the epithelial cell marker E-cadherin (Figure 1, F and G).

### Ex vivo infection of lung tissue with coronaviruses.

We began by infecting lung microtissues from 2 donors with HCoV-OC43, an endemic cause of the common cold (15, 16) that infects cells via cell membrane sialyl acid groups (17, 18). HCoV-OC43 could infect cryopreserved tissues in a dose-dependent manner, and the virus was detectable within the tissues by immunofluorescence microscopy (Supplemental Figure 2). Surprisingly, we found a marked disparity in the IL-6 transcriptional response to HCoV-OC43 in our preliminary studies between the 2 donors, with a 100-fold increase in one and no change in the other (Supplemental Figure 2).

**Figure 2:**
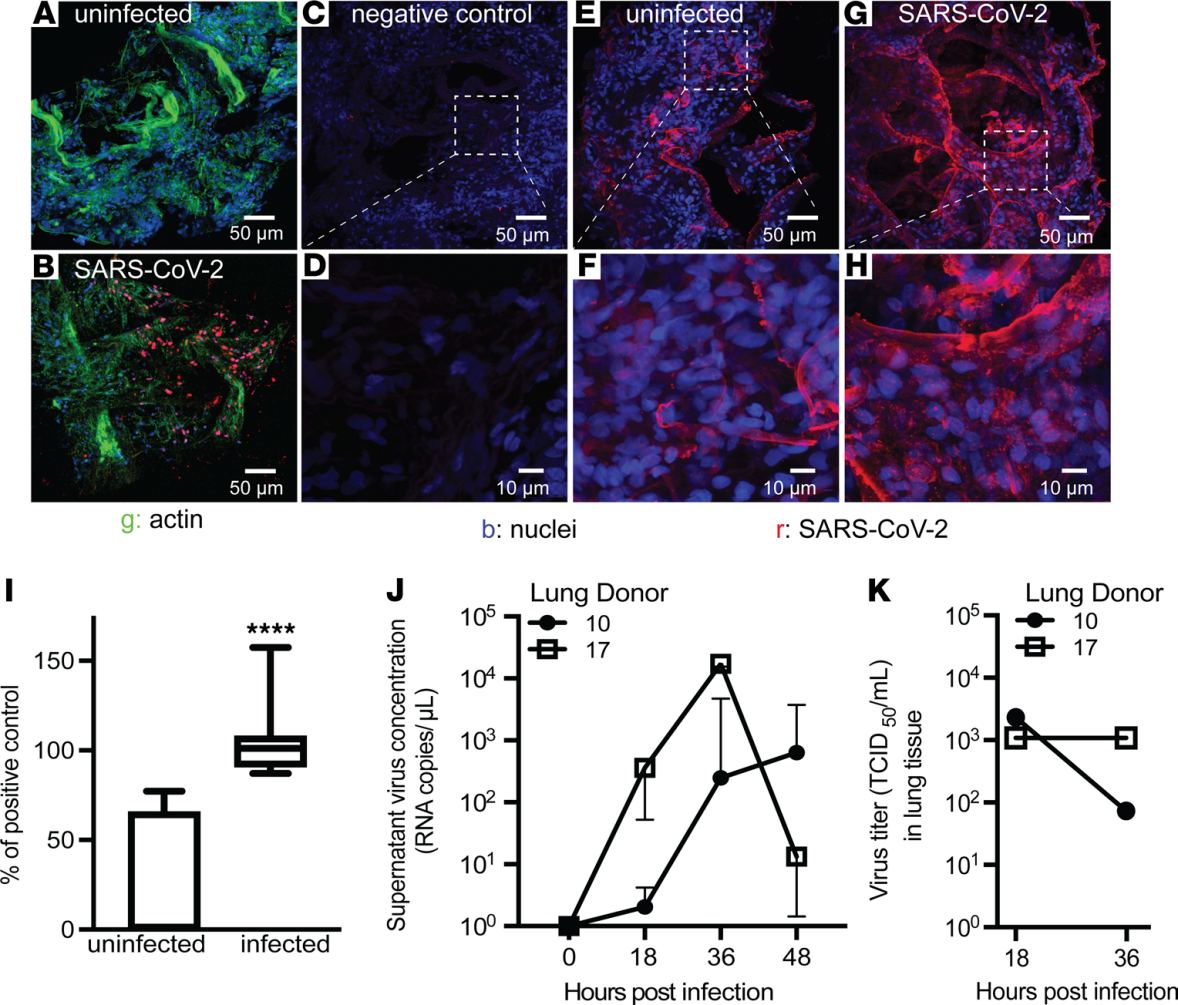
Lung microtissues can be infected with SARS-CoV-2. (**A** and **B**) Micrographs of lung tissue stained with a 1:1 mix of antibodies for spike and nucleocapsid protein to detect SARS-CoV-2 and actin. (**C** and **D**) RNA in situ hybridization of SARS-CoV-2–infected lung tissue with a negative control probe specific for the DapB gene of *Bacillus* *subtilis*. (**E**–**H**) RNA in situ hybridization of lung tissue using a probe specific for the negative strand of the subgenomic E gene of SARS-CoV-2 at 24 hours after infection. Images are representative of microscopy performed on tissue from 2 donors. (**I**) Quantitative PCR for the subgenomic E gene of SARS-CoV-2 in 16 donors at 24 hours after infection. Range of uninfected samples = 0–77.2, mean = 25.8, standard deviation = 34.6. Range of infected samples = 87.4–157.4, mean = 103.1, standard deviation = 16.7. Significance was determined by the nonparametric Mann-Whitney *U* test. (**J**) Copies of SARS-CoV-2 detected in tissue culture supernatant at the indicated time after the start of infection. Samples were infected for 12 hours with 10^4^ PFU and then washed and transferred to fresh media in a new plate. At each time point40 μL aliquots were collected. Error bars are from triplicate wells collected from each donor at each time point. (**K**) TCID_50_ assay of lung homogenate using tissue from the same donors as in **J** at 18 and 36 hours after infection. TCID_50_ was determined by counting 6 wells per donor at each dilution.

We then tested the ability of SARS-CoV-2 to infect the tissue, focusing on viral replication to demonstrate that the tissue infection resulted in production of new infective virions. We found specific staining for the spike and nucleocapsid proteins of SARS-CoV-2 within the lung tissue 24 hours after infection (Figure 2, A and B). To determine if the virus was replicating within the tissue, we next performed single-molecule fluorescence in situ hybridization with a probe specific for the negative strand of SARS-CoV-2 E gene subgenomic RNA, which is associated with viral replication, and observed a positive signal in infected lung tissue (19, 20) (Figure 2, C–H). To determine if viral replication was consistent across multiple donors, we then performed quantitative PCR for the negative strand of E gene subgenomic RNA and observed that all infected tissues produced a positive signal (Figure 2I). We confirmed that virus was shed from infected tissues by infecting samples for 12 hours and then removing the tissue from the culture plate and placing the tissue in fresh media in a new culture plate. We observed an increase in the amount of virus found in culture media, measured by the detection of nucleocapsid 1 in RNA isolated from cell culture supernatant, from 18 to 48 hours after infection compared with uninfected tissues (Figure 2J). Finally, we showed that the virus isolated from lung tissue was infectious with a 50% tissue cell culture infectious dose (TCID_50_) assay at 18 and 36 hours after infection (Figure 2K).

**Figure 3:**
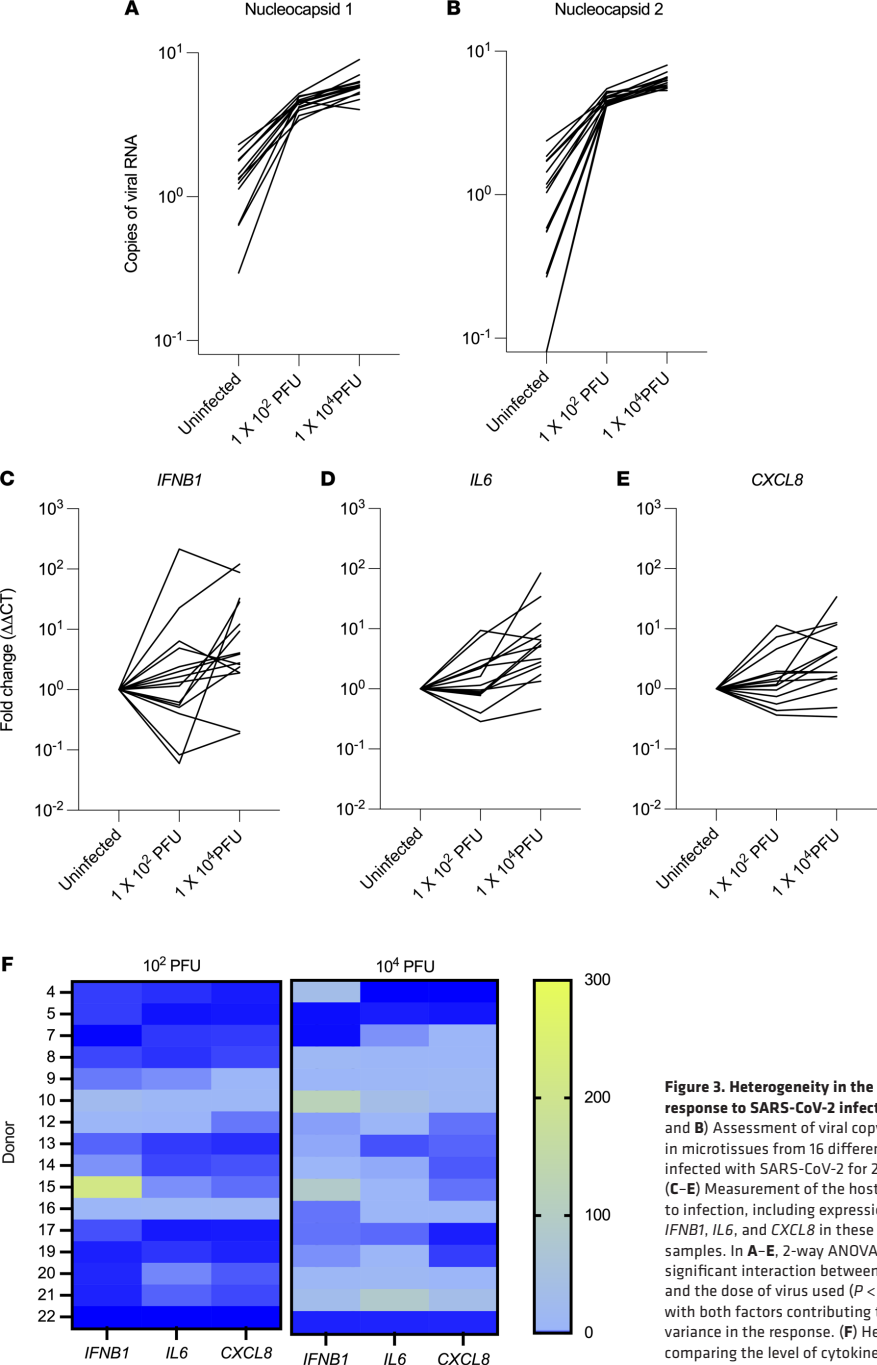
Heterogeneity in the host response to SARS-CoV-2 infection. (**A** and **B**) Assessment of viral copy numbers in microtissues from 16 different donors infected with SARS-CoV-2 for 24 hours. (**C**–**E**) Measurement of the host response to infection, including expression of *IFNB1*, *IL6*, and *CXCL8* in these same samples. In **A**–**E**, 2-way ANOVA indicates significant interaction between the donor and the dose of virus used (*P* < 0.0001), with both factors contributing to the variance in the response. (**F**) Heatmaps comparing the level of cytokines in each donor at each dose of virus.

We next sought to better assess the variance in the host response to SARS-CoV-2 infection within our tissue bank. As expected, we found an inoculum-dependent increase in viral protein transcription using 2 probes for the nucleocapsid protein that are used routinely in clinical testing (21) (Figure 3, A and B). We selected *IFNB1*, *IL6*, and *CXCL8* as markers of host response, representative of overlapping transcriptional responses to viral infection. We found highly heterogeneous responses among donors in response to the same viral inoculum, with a range of 0 to 34-fold induction in *CXCL8*, 0 to 85-fold induction in *IL6*, and 0 to 214-fold induction in *IFNB1* (Figure 3, C–E). We found no correlation between the host response and age, sex, blood type, or presence of comorbidities in this cohort (data not shown). Comparison of cytokine induction within each donor showed that most donors displayed greater induction of cytokines’ transcription with higher viral inocula (Figure 3F). Within each donor, there was a strong correlation between the induction of *IL6* and *CXCL8* in response to both low and high viral inocula (*R^2^* > 0.92), albeit with marked interindividual variability, but no correlation between the expression of *IFNB1* with either *IL6* or *CXCL8* (*R^2^* < 0.1; Figure 3F and Supplemental Figure 3).

### Antiviral therapy in lung cultures.

**Figure 4:**
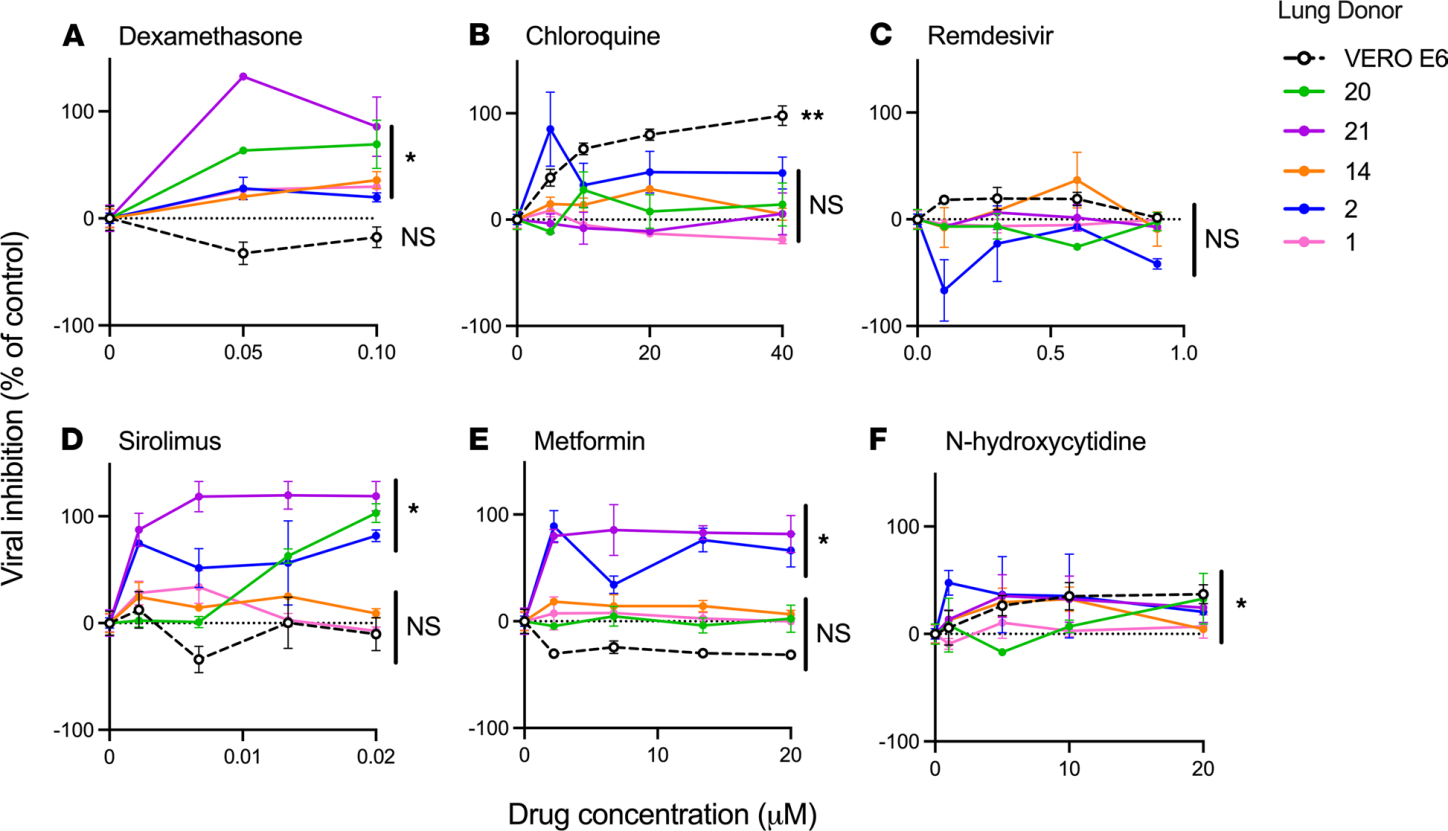
Drug treatment reduces viral titer in lung microtissues. (**A**–**F**) The percentage inhibition in viral titer for 6 different drugs in 5 different donors and the Vero E6 cell line. For DMSO-soluble drugs (remdesivir and sirolimus), the percentage inhibition was calculated using infected samples treated with an equivalent amount of DMSO. Significance was determined using a 2-way ANOVA to test for interaction between individual donors and the dose of drug. Three technical replicates were analyzed for each donor and treatment. *P < 0.05; **P < 0.01.

We reasoned that drug testing for COVID-19 relies on in vitro infections of cell lines, which may not accurately represent the response of human tissue to infection. To assess this, we next tested the effect of 6 drugs on SARS-CoV-2 viral titer in lung tissue from 5 donors and Vero E6 cells. The donors were randomly selected from our cohort (3 women and 2 men; a mean age 72). We found that dexamethasone, a drug found to improve outcomes in COVID-19 infection (19), attenuated virus titers in all donor tissues but not in Vero E6 cells (Figure 4A). In contrast, chloroquine reduced virus titers in Vero E6 cells, but not in any of the lung tissue donors (Figure 4B), consistent with prior research demonstrating that chloroquine does not inhibit virus titer in lung epithelial cells (20, 21). Remdesivir, which has been variably effective in COVID-19 in clinical trials (22–24), did not significantly affect virus titers in any of the ex vivo infections (Figure 4C).

Finally, we tested 3 investigational therapies previously predicted as potentially effective in SARS-CoV-2 infection, including sirolimus (25, 26), metformin (27–29), and β-D-N^4^-hydroxycytidine (N-hydroxycytidine) (30). We found that sirolimus was highly effective in reducing virus titer in tissues from 3 donors but did not significantly affect titers in 2 donors or Vero cells (Figure 4D). A similar heterogeneity was observed with metformin (Figure 4E). N-hydroxycytidine, an experimental drug that inhibits viral transcription (30), resulted in a significant reduction in virus titers in all tested samples (Figure 4F). These results highlight the considerable heterogeneity in antiviral host response and the effectiveness of these medications in human tissues.

### Impact of dexamethasone on lung inflammation.

Dexamethasone is a corticosteroid and proven therapy for SARS-CoV-2 infection (22, 23). The mechanism of action of dexamethasone that improves the outcome of SARS-CoV-2 infection is not empirically established but is thought to be attenuation of inflammation (24, 25).

We tested if an interventional treatment with dexamethasone was as effective in reducing virus titer in lung tissue as a prophylactic dose and observed that the delayed treatment caused a slight but nonsignificant increase in viral growth (Supplemental Figure 4).

**Figure 5:**
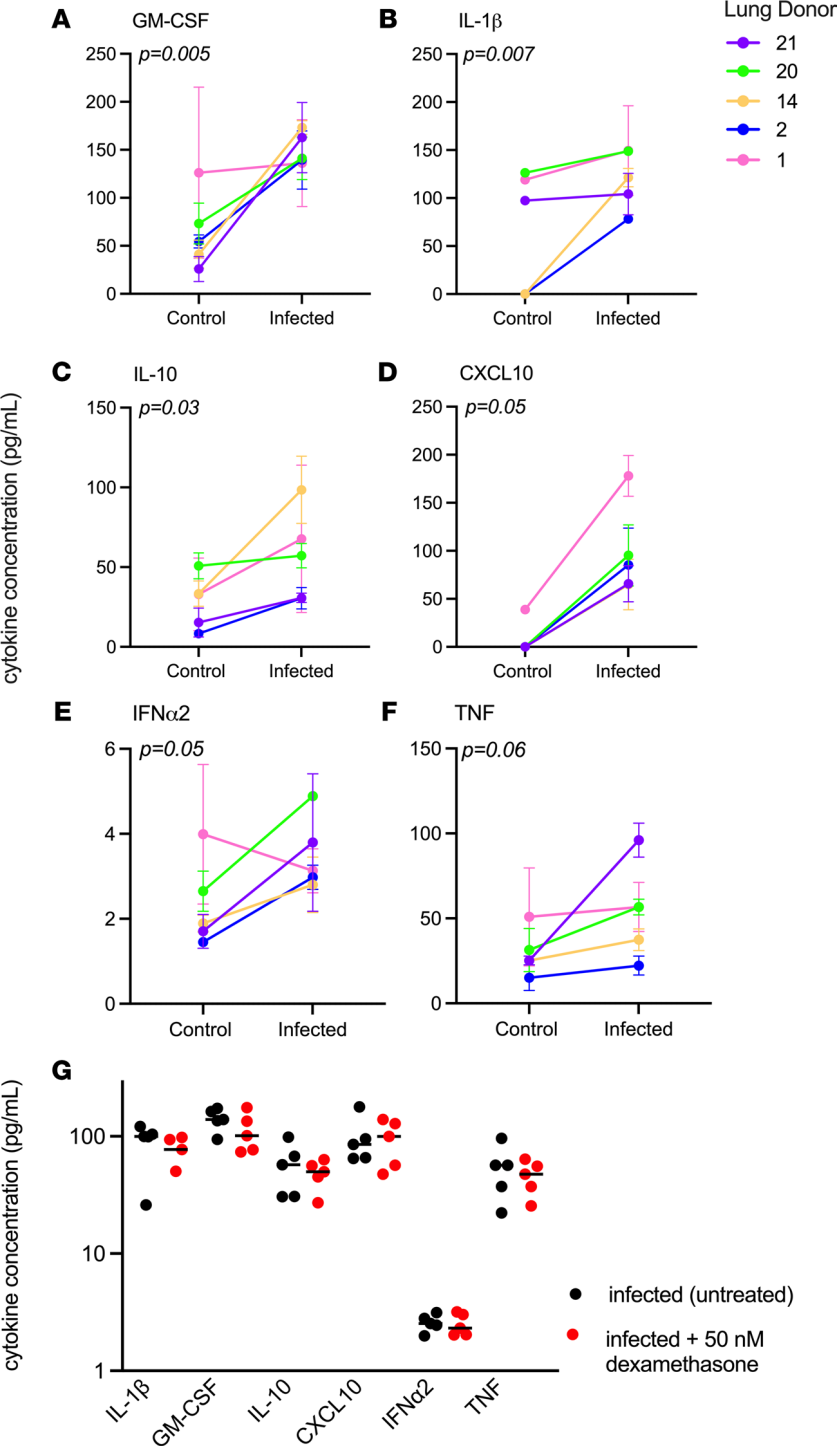
Production of inflammatory mediators is unaffected by dexamethasone treatment. (**A**–**F**) The production of inflammatory mediators is increased in lung tissue as a result of SARS-CoV-2 infection. Significance was determined with 90% confidence (*P* < 0.1) by repeated measures ANOVA. In cases where values were undetected, they were counted as 0 pg/mL. (**G**) The average cytokine value for each donor in infected tissue that was untreated or treated with 50 nM dexamethasone. Three technical replicates were analyzed for each donor and treatment.

Since we observed that dexamethasone reduced virus titer in our system, we assessed the level of cytokine production with the hypothesis that reduced inflammation may accompany a reduction in virus titer. We measured 13 soluble inflammatory mediators in the cell culture supernatant of lung tissues that were infected with SARS-CoV-2 and treated with dexamethasone, using tissue from the same 5 donors depicted in Figure 4. We observed a significant increase in IL-1β, TNF, IL-10, CXCL10, IFN-λ1, IFN-α2, and GM-CSF in infected tissue compared with uninfected controls (Figure 5, A–F). Surprisingly, none of the upregulated cytokines were reduced as a result of dexamethasone treatment in our system (Figure 5G). Of the other measured cytokines, IFN-γ, IL-12, and IL-8 were not detected, and IL-6, IFN-λ2/3, and IFN-β were not significantly increased as a result of infection at the 24-hour time point (Supplemental Figure 4).

## Discussion

The ex vivo study of human lung tissues has been hampered by difficulties in growing and maintaining 3D tissue in culture. The current technologies to achieve this, namely lung-on-a-chip, lung organoids, and precision-cut lung slices, have provided invaluable insights into our understanding of human lungs. While each of these technologies has its strengths and limitations, all are limited by the need for fresh tissues. Our study demonstrates that polyampholyte-based cryopreservation media can be used to preserve normal and diseased lung tissues in large batches. After cryopreservation, thawed microtissues displayed excellent viability, were metabolically active, and contained the major cell populations of the human lung. Our data agree with previous studies demonstrating the advantages of using polyampholyte-based media as a cryopreservative (8, 9). These lung tissues also contain extracellular matrix components that may be lacking in other models, which can be visualized in Figures 1 and 2.

A notable finding in our study was that the human tissues tested displayed similar infectability after inoculation with a given SARS-CoV-2 dose, as measured by expression of virus nucleocapsid proteins, suggesting that interindividual variability in infection is not attributable to the susceptibility of host respiratory tissues to infection (Figure 3, A and B). In contrast, infection with a given viral inoculum resulted in dramatically interindividual heterogeneity in cytokine responses of the infected tissues (Figure 3, C–E, and Figure 5). In this context, multiple studies have identified the variability in host susceptibility to COVID-19 infection, using clinical outcomes, cytokine responses, and duration and extent of viral shedding as readouts, leading to the identification of acquired risk factors and polymorphisms as risk factors for severe disease (26–32). Our data add to this literature by assessing the effect of a uniform viral inoculum between hosts, allowing us to distinguish between tissue susceptibility to infection as opposed to antiviral and inflammatory host responses.

Another important component of our work was the demonstration of antiviral drug testing using primary human lung tissues, which represents an advance over the study of drugs in the context of nonphysiologic cell lines. In this context, we confirmed the in vivo effectiveness of dexamethasone, and the ineffectiveness of chloroquine, in suppressing viral growth in human tissues. A surprising finding in our work was the lack of effect of remdesivir in both Vero E6 cells and human tissues, in contrast to prior studies in several cell lines (33–35). One potential explanation for this discrepancy is that these studies used a much lower MOI (of 0.05 to 0.2), whereas we used an MOI of 1 in our Vero cell cultures to standardize to the number of plaque-forming units to that used in our lung culture system. One study demonstrated that remdesivir exhibited potent antiviral activity in primary airway epithelial cells cultured using an air-liquid interface (33). Interestingly, this study demonstrated that remdesivir was not effective in reducing viral titer in Vero cells, similar to our findings. Overall, the difference in experimental conditions likely explains the variance in the published literature regarding the effectiveness of remdesivir in vitro.

We recognize a number of limitations in our study. First, lung tissue is currently only viable for up to 96 hours in our culture system, thereby limiting the observations regarding pathogenesis of infection to this short time frame. Second, like all in vitro systems, this culture system includes nonphysiologic features that may influence the biology of infection, nonphysiologic culture media, and absence of air. Third, the system does not capture the biology of recruited leukocytes, which undoubtedly play an important role in the evolution of lung injury in COVID-19. The absence of these cells explains the absence of detectable levels of IFN-γ and IL-12 in infected tissue because they are produced by recruited antigen-specific T cells and dendritic cells, respectively. Fourth, the sample size in our study was underpowered to detect host factors that may predispose to tissue infectibility, aberrant host response, or effectiveness of antiviral drugs and serves only as proof of principle. The use of this limited number of samples does not fully represent the range of responses to SARS-CoV-2 infection. A final limitation of our study was the inability to detect CXCL8 in cell culture supernatant despite the observed increase in transcript levels in tissue infected with SARS-CoV-2. We attribute this discrepancy to the presence of extracellular matrix components, which are known to bind CXCL8 (36) and may inhibit the detection of low levels of this chemokine. The low levels of IFN-λ1, IFN-λ2/3, and IFN-β1 may be due to the 48-hour time point, at which time much of the available cytokine may be used by cells within the culture system.

The current work suggests a number of avenues for further research. First, the ability to infect human tissues with SARS-CoV-2 ex vivo allows for a systematic study of early events in different cell types in the lung and comparison of these between different hosts, potentially leading to new insights into disease pathogenesis and heterogeneity of disease phenotype in patients. Second, the current system provides a platform for the study of other antiviral drugs in a human system, to prioritize drugs for in vivo testing or clinical trials. Third, ex vivo infection of human lung allows for mechanistic studies to define mechanisms of actions of medications. For example, the mechanisms that lead to inhibition of viral titer in response to sirolimus, metformin, or dexamethasone are unknown: while several studies have proposed mechanisms for metformin and sirolimus (37–39), dexamethasone is thought only to inhibit the host response without disrupting the viral life cycle. Our data indicate that early viral titer in lung tissue may be linked to a corticosteroid-sensitive mechanism that involves the host response to infection.

In summary, we provide evidence that human lung tissue can be utilized in vitro for the short-term study of SARS-CoV-2 infection. A lung tissue bank may also serve as a tool for the study of lung biology and drug screening beyond COVID-19 and facilitates the screening of multiple donors, in parallel, for purposes of toxicity and efficacy in primary human tissues. We envision that screening therapeutic agents in primary human tissue will take place after screening in cell lines and in parallel with testing in animal models.

## Methods

### Recruitment, sample collection, and processing.

Lung tissues were obtained from participants undergoing lung resection surgery or from allografts that underwent volume reduction before transplantation. In cases of lung resection for nodules, samples far from the site of pathology were utilized. Pleural tissue and staple lines were dissected away, and the lung was sectioned into approximately 0.5 g pieces, divided between the wells of a 24-well plate, and manually disintegrated with surgical scissors into samples with a mean diameter of 0.91 mm (range 0.40–1.5 mm) in diameter. Each well was resuspended in 1.2 mL of commercial DMSO-free cryopreservation medium (CryoSOfree, C9249; MilliporeSigma), divided into 200 μL aliquots and transferred to cryotubes containing 800 μL of additional cryopreservation media. Samples were frozen at –80°C overnight, then transferred to vapor-phase liquid nitrogen storage.

### Viral cultures.

HCoV-OC43 and SARS-CoV-2 viruses were propagated in Vero E6 cell line (ATCC CRL-1586) in advanced DMEM (12491015; Gibco, Thermo Fisher Scientific) supplemented with 10% HyClone Defined low antibody, heat-inactivated, γ-irradiated fetal bovine serum (FBS; SH30070.03IR2540; Cytiva); 1% l-alanine and l-glutamine supplement (GlutaMAX, 35050061; Gibco, Thermo Fisher Scientific); and 1% penicillin/streptomycin mixture (17-602E; BioWhittaker, Lonza), at 37°C in 5% CO_2_ in 75 cm^2^ flasks.

To inoculate the virus, media were removed from flasks with more than 80% confluent cell monolayers and replaced with 3 mL of fresh media; 1 mL of viral stock (containing 10^6^ PFU) was added to each flask; and the flask was incubated at 37°C (33°C for HCoV-OC43) in 5% CO_2_ for 1 hour, with manual rocking every 15 minutes. A “mock-infected” negative control cell culture was inoculated with 4 mL of media without virus and handled in parallel. Flasks were observed daily for development of cytopathogenic effect (CPE) and harvested after 48–96 hours of incubation when CPE had reached at least 95% of cells and approximately 25% cell detachment. The media were removed and the contents centrifuged at 2000*g* for 10 minutes at room temperature. The supernatant was then stored at –80°C overnight in 1 mL aliquots (40). Viral titer of each batch was determined via 0.9% methylcellulose plaque assay with crystal violet staining 7 days after inoculation after formalin fixation. The plaque assay protocol was modified from a previous study (41).

### Tissue culture, infection, and drug treatments.

Viral stock was thawed and diluted to the desired concentration (ranging from 10^2^ to 10^4^ PFU/mL) in commercial media (PneumaCult-Ex, 05008; STEMCELL Technologies) with 1% penicillin/streptomycin mixture (17-602E; BioWhittaker, Lonza), 50 μg/mL gentamycin sulfate (345815; MilliporeSigma), and 1.25 μg/mL ertapenem sodium (SML1238; MilliporeSigma). Ultra-low binding, flat-bottom, 96-well cell culture microplates (3474; Corning) were hydrated with Dulbecco’s PBS (DPBS) for 30 minutes at 37°C, DPBS was aspirated, and 100 μL of virus-containing media was added to each well. Biopsies were thawed for 5 minutes in the 37°C water bath, then transferred to a 15 mL tube containing 10 mL DPBS using 1000 μL wide-bore pipette tips and centrifuged at 200*g* for 1 minutes; supernatant was aspirated, and biopsies were resuspended in 1 mL PneumaCult media (described above). The concentration of biopsy samples was measured by counting the number of samples in 20 μL on a microscope slide, and 20–40 biopsies in 20 μL were added to the virus-containing wells. Each vial of biopsies provided enough biopsies for about 10 wells at this concentration. Since this culture system does not allow for enumeration of the number of cells per tissue at the outset of the experiment, we quantified the viral inocula as PFU/mL that achieved an MOI of 0.1 to 0.01 in in vitro infection of human cells (42–44). Tissues were infected with the specified PFU in 100 μL of media for 24 hours. The inoculated plate was incubated at 37°C (33°C for HCoV-OC43 infections) in 5% CO_2_ for 24 hours. Vero E6 cells used to test inhibition of viral growth were cultured to approximately 90% confluence prior to testing and then infected with 10^4^ PFU (approximating an MOI of 1) in viral growth media as described above. A total of 10^4^ PFU was used to enable a direct comparison between Vero cell cultures and lung microtissues infected with SARS-CoV-2.

To study the effect of medications on viral replication, the following drugs were used: β-D-N^4^-hydroxycytidine (9002958; Cayman Chemical), chloroquine (C6628; MilliporeSigma), water-soluble dexamethasone (D2915; MilliporeSigma), metformin (A10573; Adooq Bioscience), remdesivir (329511; Medkoo Biosciences), and sirolimus (A10782; Adooq Bioscience). To calculate percentage inhibition for remdesivir and sirolimus, which are soluble in DMSO, samples were infected and treated with an equal volume of DMSO in the absence of drug to determine the maximum signal. All other drugs were water soluble and were compared with the infected but untreated control.

### Flow cytometry.

Biopsies were resuspended in RPMI-1640 (12-167F; BioWhittaker, Lonza) with 125 ng/mL Liberase (LIBTM-RO, 5401119001; MilliporeSigma) and 50 U/mL DNase I (D5025; MilliporeSigma), agitated for 1 hour at 37°C, and then aspirated 30 times into a 1 mL syringe through an 18 G needle to form a single-cell suspension. Cells were centrifuged at 400*g* for 5 minutes at room temperature, washed twice in RPMI-1640, resuspended in flow cytometry buffer (1 mL PBS with 2% FBS and 1 mM EDTA), and filtered through 100 μm Nitex nylon mesh (57-103; Genesee Scientific). Concentration and viability were determined under a hemocytometer and trypan blue exclusion (1691049; MP Biomedicals).

Cells were washed and resuspended in 100 μL PBS and stained with a fixable viability dye (Zombie Aqua, 423101; BioLegend) for 10 minutes at room temperature, protected from light. Cells were then washed and resuspended in 100 μL buffer and stained with antibodies against various surface cell markers (Table 2). During staining, serum from humans with AB blood group (HP1022; Valley Biomedical) was added to the samples to block nonspecific binding. Surface marker antibodies were added at a concentration of 0.5 μL per 100 μL, then incubated for 20 minutes at room temperature on the orbital shaker while protected from light. After staining, samples were washed twice in flow cytometry buffer, centrifuged, and then fixed for 10 minutes in neutral buffered formalin. After incubation with formalin, samples were again centrifuged at 400*g* for 5 minutes, the formalin was removed, and then samples were washed twice with PBS. Resuspended samples were analyzed on a FACSAria II instrument (BD Biosciences) or Cytoflex (Beckman-Coulter). Unstained cell samples from each donor and compensation beads from the Invitrogen AbC Total Antibody Compensation Bead Kit (A10497; Thermo Fisher Scientific) were used to set voltages and create single-stain controls. Flow cytometry data were analyzed using FlowJo X (BD Biosciences).

### Quantification of viral titers and cytokines’ responses.

Media were aspirated from 40–60 lung microtissues or from wells containing Vero cells, and samples were washed in PBS, then placed into 500 μL of TRIzol LS Reagent (10296-028; Thermo Fisher Scientific). RNA was isolated using Zymo Direct-zol RNA MicroPrep kits according to the manufacturer’s instructions (11-330MB; Genesee Scientific) and quantified using a NanoDrop spectrophotometer (Thermo Fisher Scientific), and 30 ng was reverse-transcribed using iScript cDNA Synthesis Kit (1708891; Bio-Rad). Real-time PCR was performed using Applied Biosystems TaqMan Gene Expression Master Mix (4369016; Thermo Fisher Scientific) with predeveloped primer/probe assays from Thermo Fisher Scientific (*CXCL8* Hs00174103_m1, *IFNB1* Hs01077958_s1, *IL6* Hs00174131_m1; 433118). The ΔΔ Ct was calculated using the 18S ribosomal RNA primer/probe set (4319413E; Thermo Fisher Scientific). Nucleocapsid proteins 1 and 2 were assessed using primer/probe mixes (2019-nCoV RUO Kit, 10006713), and a standard curve was generated using the 2019-nCoV_N_Positive Control (10006625; both from Integrated DNA Technologies [IDT]).

Detection of subgenomic RNA was performed using SuperScript One-Step RT-PCR system (10928042; Thermo Fisher Scientific) with E assay probe and positive control (293417424 and 293417425; IDT) (45). At least 50 ng of RNA was used in each sample analyzed. To detect viral replication in the supernatant of infected lung tissue, samples were incubated with 1 × 10^4^ PFU of SARS-CoV-2 for 12 hours in PneumaCult and then washed twice in 5 mL of PBS. The tissue was then placed in new wells with fresh media, and 40 μL of supernatant was collected and placed into TRIzol at the specified time points.

To determine the TCID_50_ of infected lung tissue, the tissue was ground with a mortar and pestle in an assay adapted from a previous study (46). For each donor, approximately 0.4 g of lung tissue was ground in 1 mL of viral inoculation media, and the supernatant was clarified by centrifuging the sample at 3000*g* for 5 minutes. A total of 100 μL of the clarified supernatant was overlaid onto Vero E6 cells that were 90% confluent to determine the 10^0^ dilution for the assay. TCID_50_ was calculated using the Spearman and Karber formula (47) with 6 replicate wells per dilution for each donor at each time point.

Quantification of cytokine in supernatant was done using the antiviral Legendplex assay (740390; BioLegend) using a modified protocol to incorporate Biosafety Level 3 (BSL-3) practices. The assay was performed in a V-bottom plate, and all centrifugation was performed using a plate adapter for a swinging bucket rotor with safety caps. All incubations with beads were done on an orbital shaker inside a biological safety cabinet inside the BSL-3 laboratory. After the final wash, an additional 10-minute incubation step was performed with 100 μL of 4% paraformaldehyde. During this incubation, the fluid and beads in each well were transferred to a new V-bottom plate. After this step, the plate was centrifuged and the paraformaldehyde removed and replaced with 100 μL of wash buffer. The outside of the plate was sanitized and removed from the BSL-3 lab for analysis.

### Immunofluorescence staining.

Samples were fixed in 4.0% formaldehyde in PBS for 12–16 hours at 4°C, washed twice, incubated in PBS for 1 hour at room temperature, permeabilized in 0.5% Triton X-100 (X100-100ML; MilliporeSigma) for 2 hours, washed twice, and blocked with 3% bovine serum albumin in PBS for 3 hours at room temperature. Samples were then incubated overnight with conjugated antibodies at 4°C. The antibodies used in this study include E-cadherin (560062; BD Biosciences), primary rabbit anti-ZO1 (bs-1329R-A488; Bioss Antibodies), polyclonal nuclear capsid and spike SARS-CoV-2 antibodies (NB100-56683 and NB100-56578; Novus Biologicals), and HCoV-OC43 antibodies (MAB9013; MilliporeSigma). When staining for ZO1, samples were incubated in the primary rabbit anti-ZO1 antibody overnight at 4°C, followed by washing and incubating with conjugated secondary antibody (goat anti–mouse IgG, polyclonal; A21422; Thermo Fisher Scientific) against the appropriate species for 3 hours at room temperature. Antibodies against viral proteins were directly conjugated using antibody labeling kits (ab102918; Abcam). For viability staining, a live/dead kit (R37601; Thermo Fisher Scientific) with Calcein AM and BOBO-3 iodide was utilized following manufacturer protocol, and Hoechst 33342 was added to visualize nuclei.

In situ staining for subgenomic RNA was performed using RNAScope technology (48) using probe V-nCOV2019-orf1ab-sense, which recognizes SARS-CoV-2 subgenomic RNA (19) and TSA plus Cyanine 3 dye (NEL74400; Akoya Biosciences), reconstituted in molecular-grade DMSO for a final dilution of 1:150. The following modifications were made to the protocol to accommodate staining of small pieces of fixed but unembedded lung tissue: (a) Tissue dehydration was done in 1 mL centrifuge tubes in 500 μL volume. (b) All washes were done inside microcentrifuge tubes instead of on slides. (c) After incubation in 100% ethanol, the tissue was not allowed to air-dry as this would cause the tissue to stick together. Instead, the tissue was washed once in water and then incubated in 5–6 drops of RNAScope hydrogen peroxide. (d) Antigen retrieval was done in microfuge tubes incubated in a dry bath heated to 100°C with preheated buffer. (e) Tissues were stored in PBS overnight prior to counterstaining with DAPI and imaging. All lung microtissues were then imaged using a Nikon A1R HD25 confocal microscope with high-definition resonant scanner.

### Statistics.

Data were analyzed using Prism software (version 9.0 for Mac, GraphPad). Descriptive data were summarized as median and IQR. Two-sample groups were compared using the Wilcoxon rank-sum test. Comparisons of multiple groups over a range of viral inocula were performed using 2-way ANOVA with Sidak’s multiple-comparison test when all groups were of equal size or mixed effects analysis when groups differed in size. In multiple-comparison tests, multiplicity-adjusted *P* values (Dunnett’s test) are reported. Linear correlations between variables were assessed using Pearson’s coefficient. *P* < 0.05 was considered statistically significant.

### Study approval.

The study was performed in accordance with the Declaration of Helsinki, under a protocol approved by the University of Florida Institutional Review Board (IRB202000920) after written informed consent was received from participants.

## Author contributions

MAS, YS, ZD, DN, and BM wrote the manuscript. MAS, YS, ZD, JCL, DN, JU, RS, and BM performed experiments associated with this manuscript. MAS, YS, ZD, DJO, RFC, WGS, and BM all contributed to experimental design and data analysis. DN, JU, RS, JAL, TNM, and WGS provided reagents essential for these studies. MAS, YS, ZD, DN, JU, and RS acquired data for these studies.

## Supplementary Material

Supplemental data

## Figures and Tables

**Table 1:**
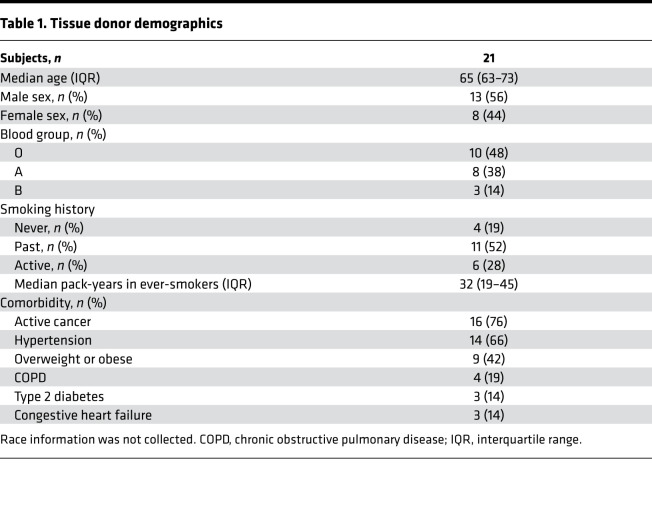
Tissue donor demographics

**Table 2:**
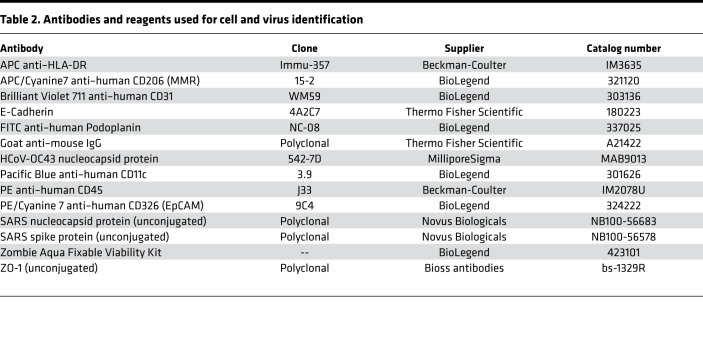
Antibodies and reagents used for cell and virus identification

## Version 1 08/06/2021

In-Press Preview

## Version 2 09/22/2021

Electronic publication
