# Supplementary material for: Ex vivo SARS-CoV-2 infection of human lung reveals heterogeneous host defense and therapeutic responses
Source: JCI Insight. 2021 Sep 22;6(18):e148003. doi: 10.1172/jci.insight.148003 (PMC8492301; doi:10.1172/jci.insight.148003)
Supplement: Supplemental data [file jciinsight-6-148003-s020.pdf]

Figure S1

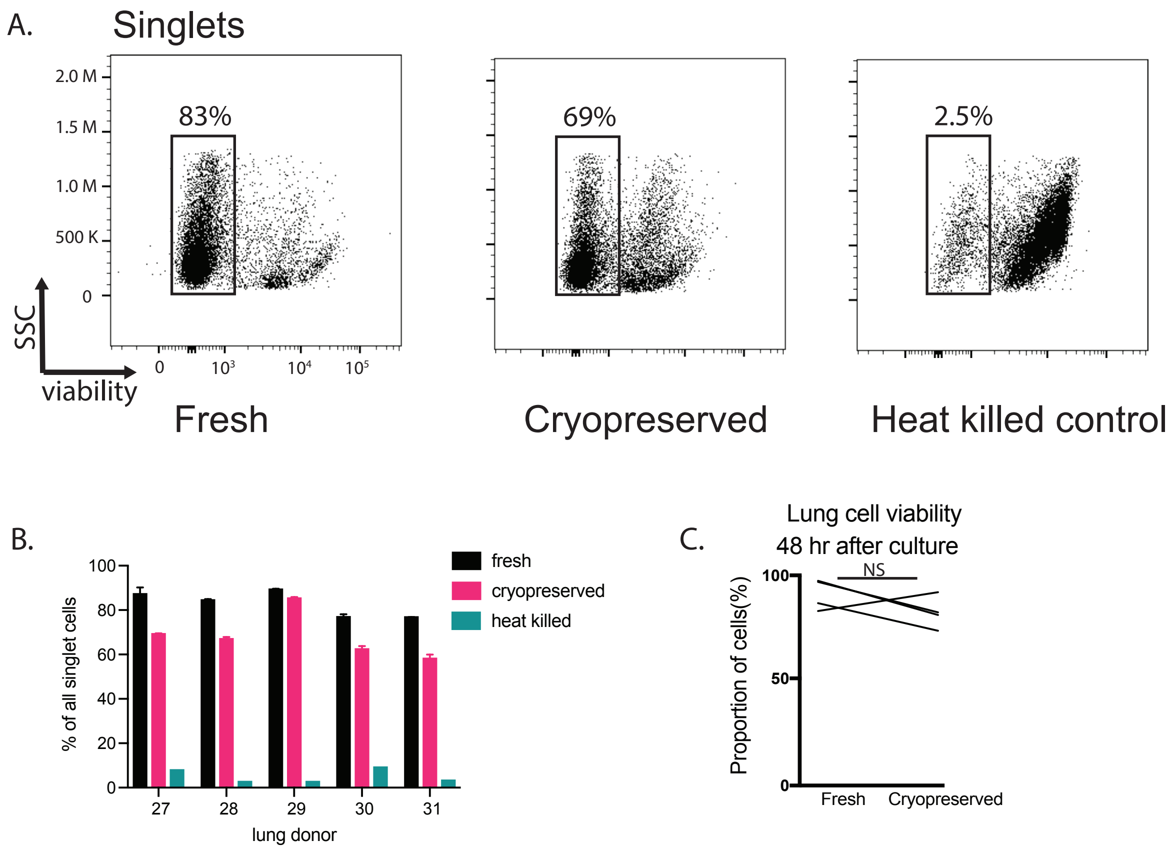

Figure S1: Lung viability after 48 hours of culture. A) An example of the gating strategy used for assessing viability in lung flow cytometry samples, including the use of a heat-killed control. B) The same graph as depicted in figure 1b, including data on heat-killed control samples for each donor assessed. C) Graph representing 4 matched samples from the same donor. Each sample consists of over 40-60 microtissues cultured for 48 hours. p=0.25 as determined by a paired nonparametric test.

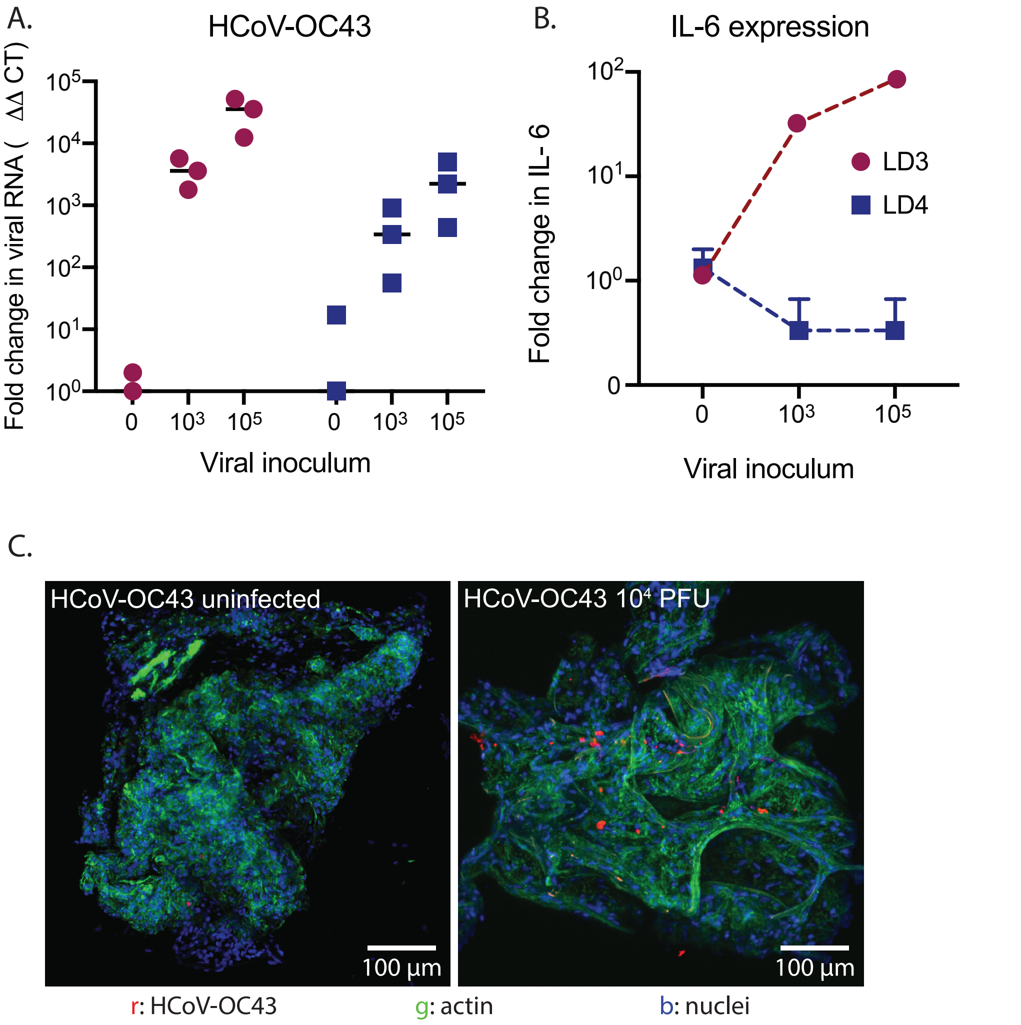

Figure S2: Lung microtissues can be infected with coronavirus.
A) Lung microtissues were infected with the specified PFU of HCoV-OC43 for 24 hours. The presence of virus in tissue was assessed by qPCR. B) The expression of IL6 in the same samples as used in A. C) Micrographs of lung tissue stained with antibodies to HCoV-OC43 and actin.

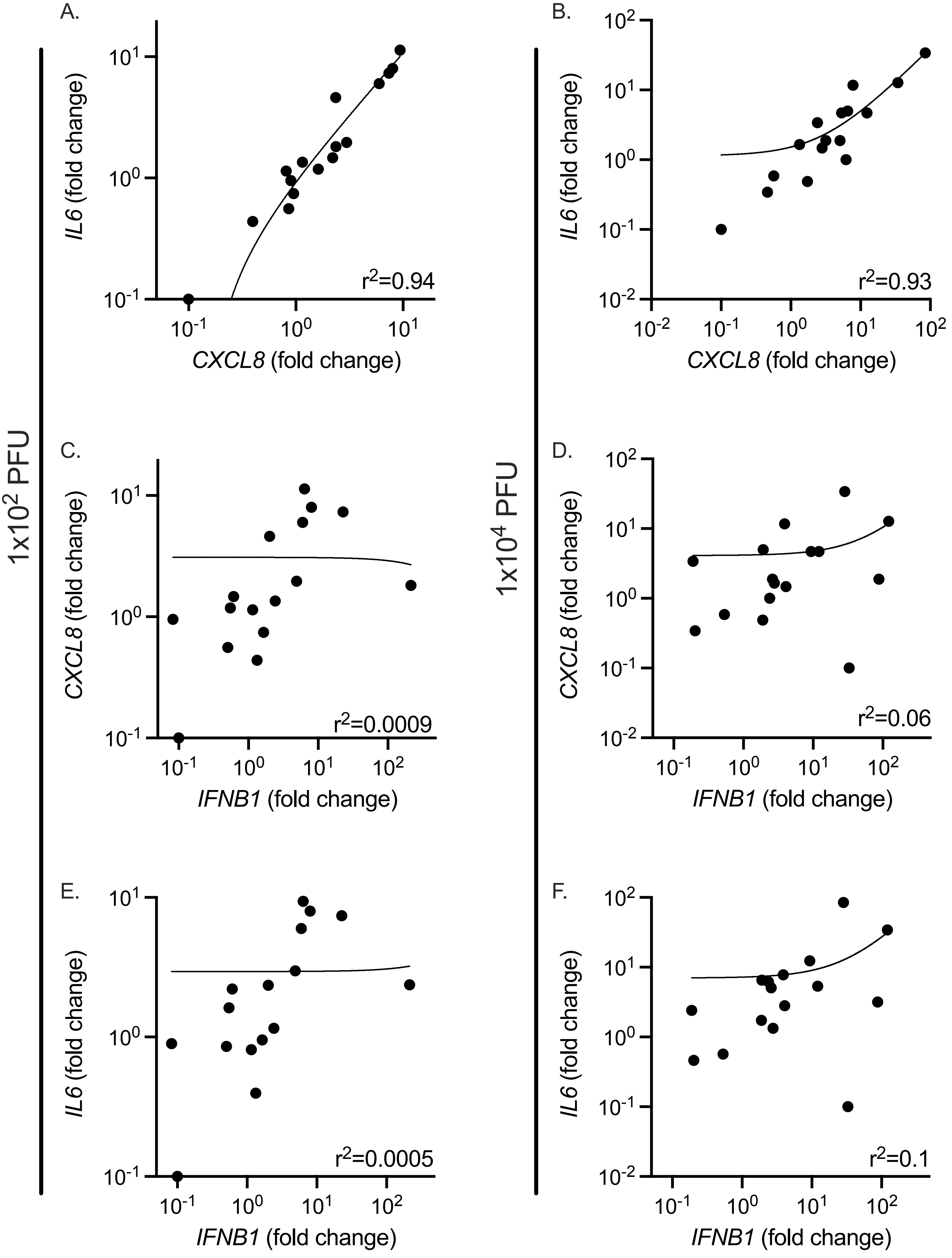

Figure S3: Correlation in host response to SARS-CoV-2. A-E) The correlation between the production of IL-6, CXCL8 and IFN-beta in each donor was assessed by linear regression for two doses of virus. R squared values are depicted in the bottom of each graph.

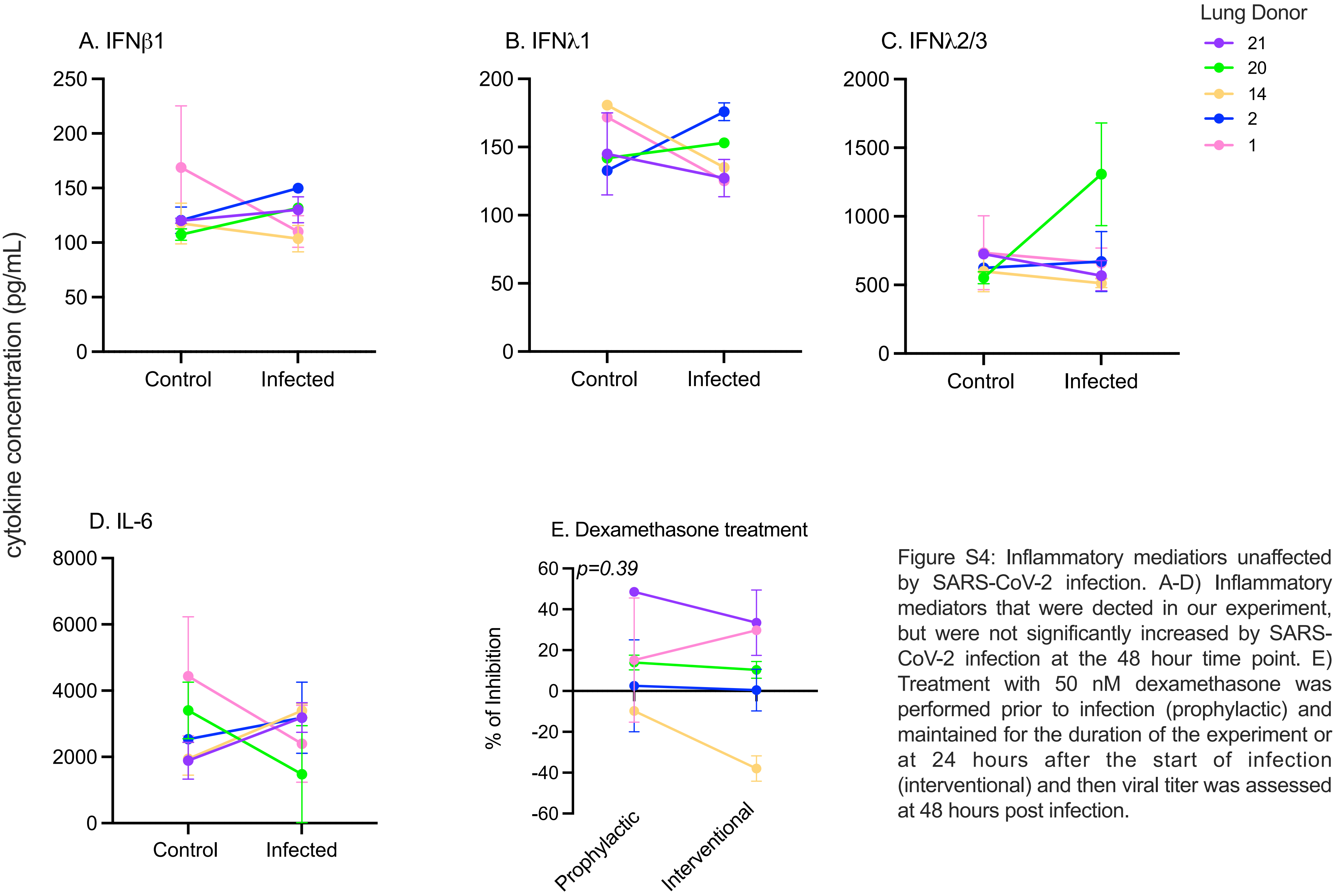

Figure S4: Inflammatory mediatiors unaffected by SARS-CoV-2 infection. A-D) Inflammatory mediators that were dected in our experiment, but were not significantly increased by SARS-CoV-2 infection at the 48 hour time point. E) Treatment with 50 nM dexamethasone was performed prior to infection (prophylactic) and maintained for the duration of the experiment or at 24 hours after the start of infection (interventional) and then viral titer was assessed at 48 hours post infection.
